# On the through-process texture evolution assessment in grain oriented Fe-3 wt% Si steel produced by a novel directional inoculation technique

**DOI:** 10.1038/s41598-021-84360-0

**Published:** 2021-03-01

**Authors:** Snehashish Tripathy, Sandip Ghosh Chowdhury

**Affiliations:** 1 Academy of Scientific and Innovative Research (AcSIR), Ghaziabad, 201002 India; 2 Materials Engineering Division, CSIR-National Metallurgical Laboratory, Jamshedpur, 831007 India

**Keywords:** Metals and alloys, Materials science, Materials for energy and catalysis, Structural materials

## Abstract

A novel directional inoculation technique has been designed to cast thin slab ingots containing Goss (or near Goss) oriented components in the as cast microstructure under the combined effect of oriented nucleation and oriented growth. The same has been targeted so as to retain Goss orientations and simultaneously develop γ fiber components (ranging from {111}<$1\overline{1}0$> to {111}<112>) during hot rolling. The designed scheme of directional inoculation achieved oriented nucleation by the effect of exogenously added soft magnetic inoculants under magnetic field and oriented growth by the effect of fast cooling rates prevailing in the mould. The choice of 65Fe–35Co (wt%) system as soft magnetic inoculants was made taking into account the similarity in crystal structure and lattice parameter. The chemically synthesized inoculants under the effect of external magnetic field during solidification were able to exhibit directional inoculation. Variation in the cast microstructure and microtexture by varying the extent of inoculant addition was studied by EBSD technique. The ingots cast under different conditions were subjected to a designed hot rolling schedule and the through process microstructural and microtextural evolution was assessed. It was observed that fine equiaxed grains with initial cube orientations in the as cast structure could lead to the most desirable microstructural as well as microtextural gradient in the hot band.

## Introduction

Fe–3 wt% Si steels, commonly used in the form of cold rolled and multistage annealed sheets for transformer cores, constitute a special class of soft magnetic materials. The steel possesses a strong and sharp Goss texture ({011}<100>) after abnormal grain growth (also termed as secondary recrystallization) within a deviation of 7° (for Conventional Grain Oriented grade-CGO) and 3° (for High Permeability grade-HGO)^1–4^. The efficacy with which the strength and the sharpness of the Goss texture is attained during the abnormal grain growth step has been attributed to the presence of a suitable precursor microstructure formed at post primary recrystallization and decarburization (and nitriding) steps. This microstructure must consist of Goss oriented grains (to act as potential nuclei for abnormal grain growth), favourable surrounding texture components (in order to facilitate selective grain boundary mobility) and homogeneous distribution of inhibitors (to pin other orientations from growth)^5–21^. Given a particular type of inhibitor and its corresponding distribution, the selective growth of Goss grains has been shown to be highly dependent upon the surrounding texture in the primary recrystallized state^8,22–25^. This surrounding texture decides the statistical frequency of the grain boundary charcter which the Goss grains would experience during the abnormal grain growth phenomena^8,15–22^. The general inference from these previous studies is that, the γ-fiber components ({111}<$1\overline{1}0$> to {111}<112>) and some of the α-fiber components (especially {411}<148>) are most favourable for the selective growth of Goss oriented grains during abnormal growth^8,9,14,15,17,23,25–27^. On the other hand, these particular γ- and α-fiber components have been experimentally shown to develop from Goss oriented grains only, when subjected to plain strain compression through cold rolling in both single crystal as well polycrystalline specimens^28–32^. Previous studies have also shown that γ- and α-fiber components (like {111}<$1\overline{1}0$> and {001}<110>) are the stable end orientations under plane strain compression (PSC) and would therefore, get retained during cold rolling, once formed^31,33^. The precursor Goss grains which can rotate to yield γ- and α-fiber components during cold rolling have been in turn confirmed to develop during the process of hot rolling of the steel^31–34^. Thus, it can be inferred that the entire process of texture evolution in grain oriented (GO) steels is based upon texture memory and inheritance, apart from the effect of inhibitors and other topological factors in the primary recrystallized material.

In order to have better control over the texture evolution and to reduce the overall complicacy of the conventional process, strip casting has been extensively explored for grain oriented Si steel. The cast strips are cold rolled (post optional hot rolling or annealing) followed by primary and secondary annealing^35–42^ to yield sharp Goss texture. It has been shown that fine recrystallized grains and a strong γ-fiber texture after primary annealing, yield better strength and sharpness of Goss texture in the thinner sheets of Fe-Si steels^40,41,43^. The extensive research on through process texture evolution has certainly led to the development of optimized process schedules for strip casting based GO steel. However, the quality in terms of magnetic properties (which comes from the inherent strength and sharpness of Goss texture) of the secondary recrystallized sheet is yet not at par with the conventionally produced CGO/HGO grades. The primary reason for this is the absence of the hot rolling step in most of the processing schedule of strip cast products. As already mentioned above, the hot rolling step plays a crucial role in the inception of Goss grains in the GO steels^31,32,34^.

The present investigation is therefore, attempted to combine the advantageous effects of hot rolling with strip casting by designing a novel thin slab casting route (where the cast thickness is ~ 15 mm). The target was to enhance the fraction of Goss texture in the hot band; these components not only ensure the presence of the same as potential nuclei (after primary recrystallization) but also the subsequent cold rolling and annealing would enhance the requisite (γ- and α-fiber) texture components at the abnormal grain growth step. In order to enhance the fraction of Goss oriented grains in the hot band, a strategy for enhancing the same in the as cast product itself was devised. This was attempted with the understanding that, being the stable orientation under shear deformation during hot rolling, the Goss grains would be inherited from the as cast texture to that of the hot band. Further, under plane strain compression prevailing at the mid layer of the specimen during hot rolling, the as-cast Goss grains would yield various γ- and α-fiber components which can be retained during cold rolling; this will create a favourable surrounding for abnormal grain growth.

For obtaining Goss components in the as cast product a novel directional inoculation technique was conceptualized in the present work wherein the advantages of both oriented nucleation as well as oriented growth would be derived. The oriented nucleation was provided by designed soft magnetic inoculants in the presence of magnetic field and oriented growth was facilitated by the faster cooling rates during solidification. The comparison of through process texture evolution between the directional inoculation based hot band and the thin slab casting based hot band will be carried out. This will help in understanding the correlation of initial texture and microstructure of the cast product with the one obtained after hot rolling.

## Experimental procedure

### Design of directional inoculation technique

The technique of directional inoculation was aimed at enhancing a particular orientation in the as-cast structure i.e. {110}||ND and <001>||RD in the present case. For obtaining a specific texture during diffusional phase transformation (especially in solidification), both oriented nucleation as well as oriented growth is required. The combination of these two phenomena can lead to a strong texture in the product phase. This has been achieved in the present work by devising a strategy addressing each of the two aforementioned aspects independently.

#### Oriented nucleation aspect

**Figure 1:**
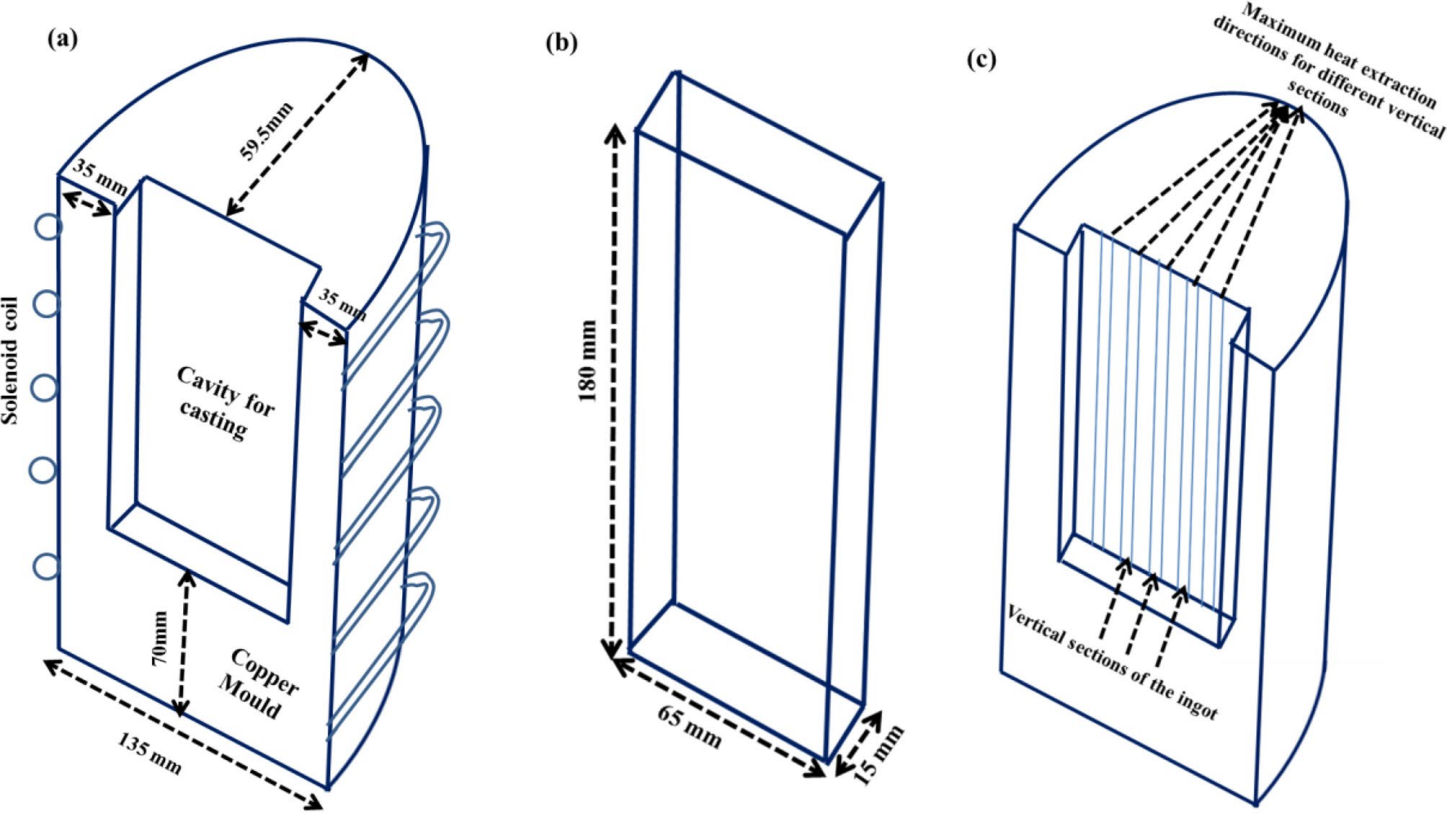
Schematic representation of the (**a**) axisymmetric view of the copper mould; (**b**) slab ingot cast within the copper mould; (**c**) maximum heat extraction direction for different sections of the ingot.

A combination of inoculants and a suitable external stimulus have been employed to control the initial orientations of the nuclei forming during solidification. Hunter et al.^44^ have adopted such a technique for ferritic stainless steels, wherein TiN inoculants are formed in-situ and they lead to oriented nucleation of the crystals from melt followed by oriented growth owing to the faster cooling rate through the copper substrate^44^. Thus, it is clear that the combination of inoculants and an external stimulating field (in the case of Hunter et al.^44^, it was density difference) can lead to oriented nucleation during initial stages of solidification. Inoculation in steels by carbides, nitrides and other mineral particles has been extensively investigated in the past^43,45^. However, none of the used inoculants are soft magnetic in nature and therefore, may hamper the final magnetic properties of the GO steel. It therefore, becomes necessary to design soft magnetic inoculants for the present work, having good lattice correspondence with the δ-ferrite phase nucleating during solidification. The above two requirements give way for the choice of Fe based alloys/compounds for the purpose of inoculation in the present work. The additional advantage of Fe based inoculants is the easy magnetization axis of <100> ^46^, which can be aligned along the RD by application of an external magnetic field. However, in order to be effective for <100>||RD oriented nucleation, these Fe based inoculants have to be magnetically susceptible at higher temperatures. It is evident from the literature that amongst the Fe based soft magnetic alloys, Fe–Co systems tend to possess highest Curie temperatures^47^. Therefore, based upon the aforementioned requirements of the inoculants and the inferences drawn from various research works, in the present investigation 65Fe–35Co (wt%) system was chosen for the purpose of directional inoculation under external magnetic field. The melting point of this inoculant (~ 1500 °C) lies above that of Fe-Si alloy used in the present investigation (~ 1480 °C). The solidification of the melt has been carried out in thick copper mould sections which results in faster heat extraction. With higher cooling rates, the solidification gets initiated at the inoculant surfaces before the temperature of those inoculants become higher than its Curie point. A cylindrical copper mould, the schematic of which is given in Fig. 1a, has been prepared with the purpose of providing fast cooling rate to the thin slab ingot cast within it. The cast ingot typically had a dimension of 180 mm × 65 mm × 15 mm as schematically given in Fig. 1b. Under axisymmetric considerations, the maximum thickness of the copper mould (and thus the maximum heat extracting section) for an ingot thickness of 7.5 mm is 59.5 mm (almost 8 times). This thickness of the copper mould is expected to render rapid extraction of heat in the initial stages of solidification. However, it should be noted here with regards to direction of maximum thickness of the mould (adjacent to ingot), that for all the sections of the ingot except the centreline, thicker portions of the mould lie at an angle to the ND. This is due to the cylindrical geometry of the mould and flat geometry of the cavity/ingot. This is schematically shown in Fig. 1c where the cavity/ingot has been divided into vertical sections and the corresponding maximum heat extraction direction (depending upon the thickness) for each section has been highlighted.

The magnetic field acting as an external stimulus to orient the inoculants was facilitated by fabricating a solenoid coil and a DC power supply unit. The mould was placed inside the solenoid coil as a core such that the solidifying steel ingot lied at the exact center of the solenoid, thereby experiencing a steady state magnetic field of ~ 20mT (as measured by Gaussmeter) when the current supplied from the source is 200A. The provided magnetic field although might seem to be very feeble, yet it is good enough to affect and align the inoculant particles, which has been confirmed by checking its effect on the particles when placed inside the mould cavity.

#### Oriented growth aspect

From the previous studies on strip cast structure, it is evident that a strong {100}|| ND (normal direction) texture is attained in the columnar zone. This is attributed to the higher rate of heat extraction in such solidification process^38,39,48–50^. The crystallographic planes with highest accommodation factor grow opposite to the heat extraction direction and outrun other misaligned orientations, thereby attaining a selective texture component during growth^51^.

The fast cooling rates provided by the copper mould are expected to lead to a positive temperature gradient ahead of the solid front and subsequent columnar growth with {100}||ND texture. However, the target texture component for as cast structure in the present study was {110}||ND and <001>||RD rather than {100}||ND texture. The crystals growing with {100}||ND, might not possess <001>||RD orientation. Therefore, in order to align the crystals with <001>||RD, oriented nucleation becomes essential, which has been uniquely devised as mentioned earlier, in the present work.

### Inoculant synthesis

The techniques adopted in the past for the synthesis of Fe–Co alloys vary from mechanical milling to diode sputtering^52–54^. However, for the present work a synthesis route is required which can render higher purity and control over the morphology of the inoculant particles such that size, surface area and volume of the particles can be optimized. For this purpose a technique of preparing Fe–Co alloys by reduction of Fe and Co containing salts has been adopted^55^. Herein, the sulphate salts of Fe and Co were taken in a ratio of 2:1 (since the targeted Fe:Co ratio in the alloy is ~ 2:1) and reduced by a solution of sodium hydroxide (NaOH) and hydrazine (N_2_H_4_) at ~ 90 °C. The reduced product particles were filtered and dried in vacuum followed by morphological and structural characterization. The structural characterization was carried out using Bruker D8 Discover X-Ray Diffractometer having Cu Kα source. The scan was carried out for a 2θ range of 40–120° with an increment of 0.02° and dwell time of two seconds per step. Materials Analysis Using Diffraction (MAUD) software was used to analyse the XRD pattern for lattice parameter and crystallite size details. The instrumental Caglioti parameters were determined by performing a scan on standard Si powder and fitting the standard Si pattern with it through MAUD. The instrumental correction for broadening, asymmetry and peak profile was done using these Caglioti parameters. The morphological and spectroscopic details were characterized in FEI Nova Nano FEG SEM attached with an Energy Dispersive X-Ray Spectroscopy detector. For the purpose of analysing the composition of the particles, the accelerating voltage was maintained in the range of 5–10 kV, so as to minimize the depth of interaction beyond the particle thickness.

### Alloy processing

Alloys of composition within the range given in Table 1 were prepared using air induction furnace and cast in the designed copper mould. The various conditions under which different casting of the slab ingots were carried out have been listed in Table 2. The first ingot (Cast 1) was cast to assess the effect of fast cooling rate alone upon the cast structure and texture, whereas the second ingot (Cast 2) was cast in the same copper mould along with the presence of external magnetic field. Three more ingots (Cast 3–5) were cast in the copper mould along with addition of 0.3, 0.5 and 0.8 wt% (of the ingot) of Fe–Co inoculants. These castings were done in the presence of external magnetic field as well so as to have the effect of directional inoculation during solidification in these ingots. The inoculants were wrapped in laboratory grade aluminium foils and half of it was placed in the mould prior to pouring of the liquid metal and rest half was added during the time of pouring. It was expected that the particles would be circulated throughout the cavity owing to the liquid metal thermo-solutal convection during pouring. Further specimens of dimension 80 mm × 40 mm × 15 mm were taken from the ingot and subjected to reheating at 1200 °C for 3 h followed by single stage hot rolling in laboratory scale two high hot rolling mill to a final thickness of ~ 2 mm. The reason for selection of 1200 °C as the reheating temperature has been given elsewhere^56^. The design of the hot rolling schedule was also based upon the inferences drawn from the previous works regarding the effect of draft schedules on the microstructural, textural and magnetic properties^57–59^. The hot rolling schedule consisted of 3 passes: the first pass consisted of a reduction level of ~ 60% so as to yield dynamically recrystallized grains and fine distribution of precipitates (MnS) during the subsequent interpass time. The second pass consisted of a moderate reduction level of ~ 40% with the aim of attaining dynamic and static (in the subsequent interpass time) recrystallization at the surface whereas recovered/static recrystallized grains at the center. The third pass of ~ 45% was given with the aim of producing dynamically recrystallized grains again at the surface while recovered grains are obtained at the center. After the hot rolling, the hot band was air cooled to room temperature.

**Table 1:** Composition range of the cast ingots prepared in the present work.

Elements	C	Si	Mn	S	Fe
Content (wt%)	0.04–0.06	3.1–3.2	0.08–0.1	0.008–0.01	Balance

**Table 2:** Casting conditions for various slab ingots.

Casting identification	Conditions of casting
Cast 1	Fast Cooling
Cast 2	Fast Cooling + Magnetic Field
Cast 3	Fast Cooling + 0.3 wt% inoculants + Magnetic Field
Cast 4	Fast Cooling + 0.5 wt% inoculants + Magnetic Field
Cast 5	Fast Cooling + 0.8 wt% inoculants + Magnetic Field

### Microtexture characterization

The microtexture characterization of both as cast and hot rolled products were carried out in the FEI Nova Nano FEG SEM equipped with TSL DigiView III EBSD detector. For acquiring the texture of the as cast structure, a minimum of 1.5 mm × 1.5 mm area in the ND-TD section at the center of the ingot was scanned whereas for the hot band specimens, through thickness areas in the ND-RD section were scanned. The samples for microtexture were prepared by standard metallographic techniques. The data acquisition was carried out at an accelerating voltage of 20 kV with a total specimen tilt of 72°. The analysis of the acquired microtexture data was carried out using TSL OIM 7.0 software. The microtexture is represented in the present work by Inverse Pole Figure (IPF) maps depicting the crystallographic plane perpendicular to ND and Orientation Distribution Functions (ODF) computed by the series expansion (rank = 22) (Bunge) method. The ODFs are sections of the Euler space represented by ϕ_1_, ϕ and ϕ_2_ axes, of which the constant ϕ_2_ sections of 0° and 45° are used for the present work.

## Results and discussion

### Fe–Co inoculants

**Figure 2:**
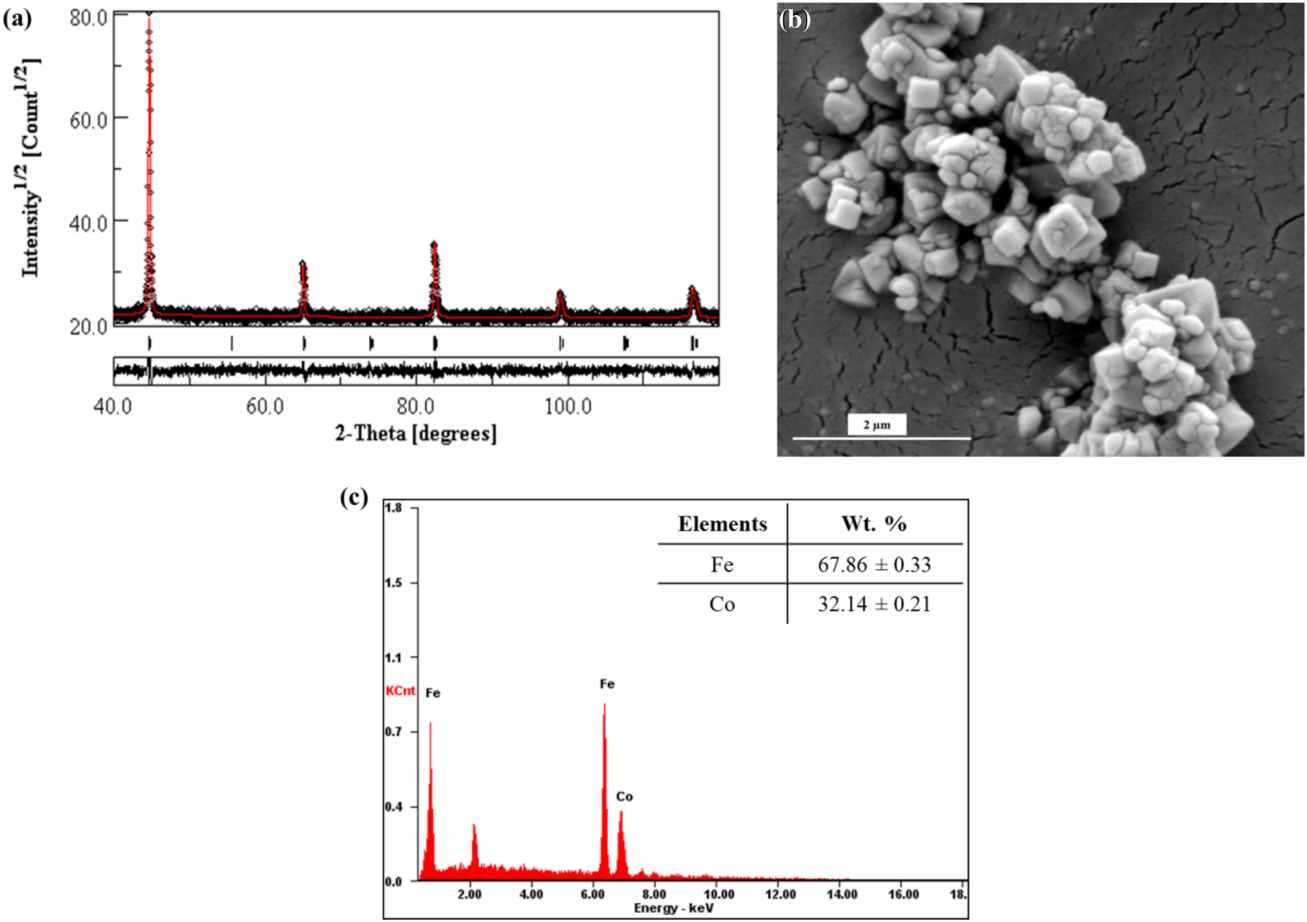
(**a**) XRD pattern of the Fe–Co alloy synthesized in the present work (peak profile fitting carried out using MAUD); (**b**) SEM micrograph of the Fe–Co particles and (**c**) composition of the particles as acquired by EDS.

The XRD pattern of Fe–Co particles is given in Fig. 2a. It also shows the standard powder diffraction pattern of Fe–Co system (with Space Group *Pm* $\overline{3}$ *m*) fitted to the experimental pattern using MAUD software along with the residual of the fitting. The standard pattern of a primitive cubic crystal system is selected because the Fe–Co particles synthesized in the present work are expected to be an ordered phase having a primitive structure owing to the composition and temperature of synthesis^47^. The lattice parameter of the synthesized Fe–Co alloy was determined to be ~ 2.87 Å. The approximate lattice parameter of Fe-Si system in BCC structure is 2.86 Å. Thus, it can be inferred that in terms of lattice registry, the inoculants must be effective. The crystallite size of the particles determined from XRD is ~ 0.30 µm. The SEM micrograph given in Fig. 2b shows the faceted cubic morphology of the particles with an average particle size of ~ 0.30 µm. This indicates that the particles are single crystals and thus the faces of the cubic morphology can be inferred as {100} crystallographic planes of the Fe–Co cubic crystal.

Further, this leads to the inference that these {100} crystallographic planes of the inoculant exposed at the particle surface would act as a substrate for the nucleation of {100} planes of the solidifying crystals on it. The composition of the Fe–Co powder synthesized in the present work and as measured using EDS has been given in Fig. 2c, which confirms the formation of ~ 68 wt% Fe–32 wt% Co alloy.

### Microstructure and microtexture of the slab cast ingots

**Figure 3:**
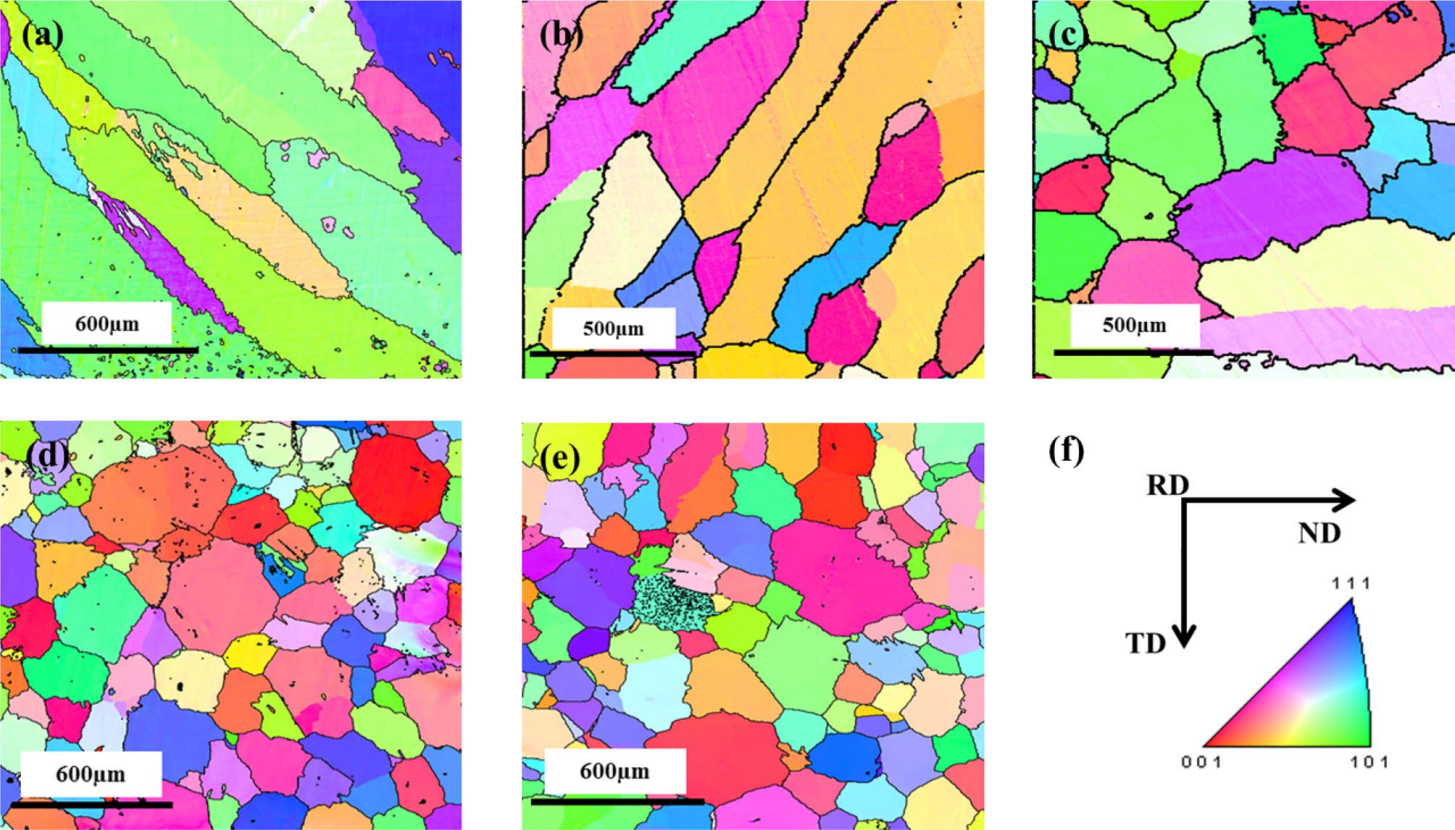
IPF maps of (**a**) Cast 1, (**b**) Cast 2, (**c**) Cast 3, (**d**) Cast 4, (**e**) Cast 5 and (**f**) directions of the slab ingot with respect to the scanned surface.

The IPF maps of the ND-TD section for Cast 1–5 are given in Fig. 3a–e, respectively. Figure 3f shows the directions of the slab ingot with respect to the scanned sections. The texture represented in the present work is in terms of {hkl}<uvw> , where <hkl>||ND and <uvw>|| RD.

It can be observed from Fig. 3a, b that the cast structure is dominated by coarse columnar grains in the absence of any inoculants, whereas Fig. 3c–e depict refined equiaxed grains with the addition of inoculants. The inoculants were thus, found to be effective in its role of refining the microstructure, as observed from the IPF maps. The IPF maps of Fig. 3a, b also depict that the columnar grains are aligned at an angle to the ND (close to 45°). These long columnar grains aligned at an angle to the ND confirm the expected difference in the maximum heat extraction direction along the TD of the ingot as mentioned in “Oriented nucleation aspect” section and Fig. 1c. The direction of heat extraction is thus inferred to depend upon the direction of maximum thickness of the mould adjacent to the ingot.

**Figure 4:**
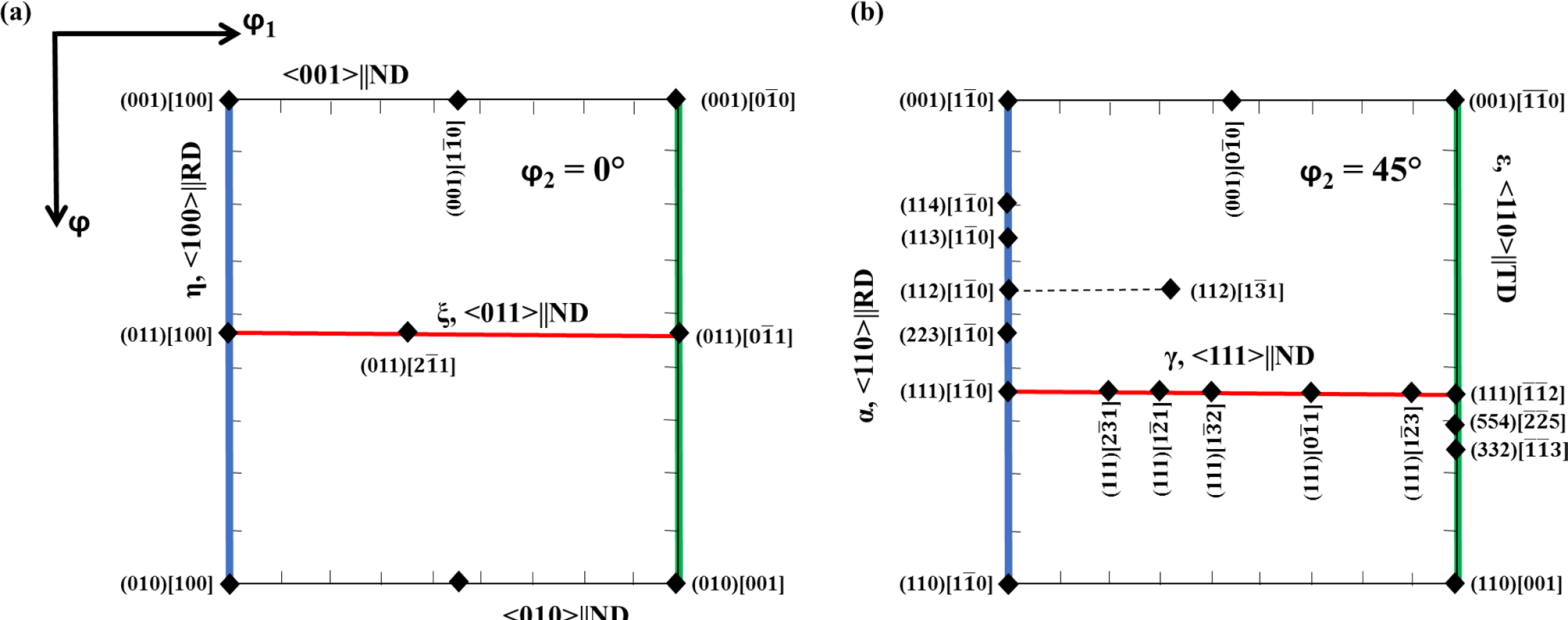
(**a**) φ_2_ = 0° section and (**b**) φ_2_ = 45° section of the Euler space along with some common orientations and fibers that occur in BCC metals and alloys.

Before proceeding on to the analysis of as cast microtexture, the standard ODF sections of φ_2_ = 0° and 45° of the Euler space along with some common orientations and fibers that occur in BCC alloys are presented in Fig. 4a, b, respectively^33,60^.

**Figure 5:**
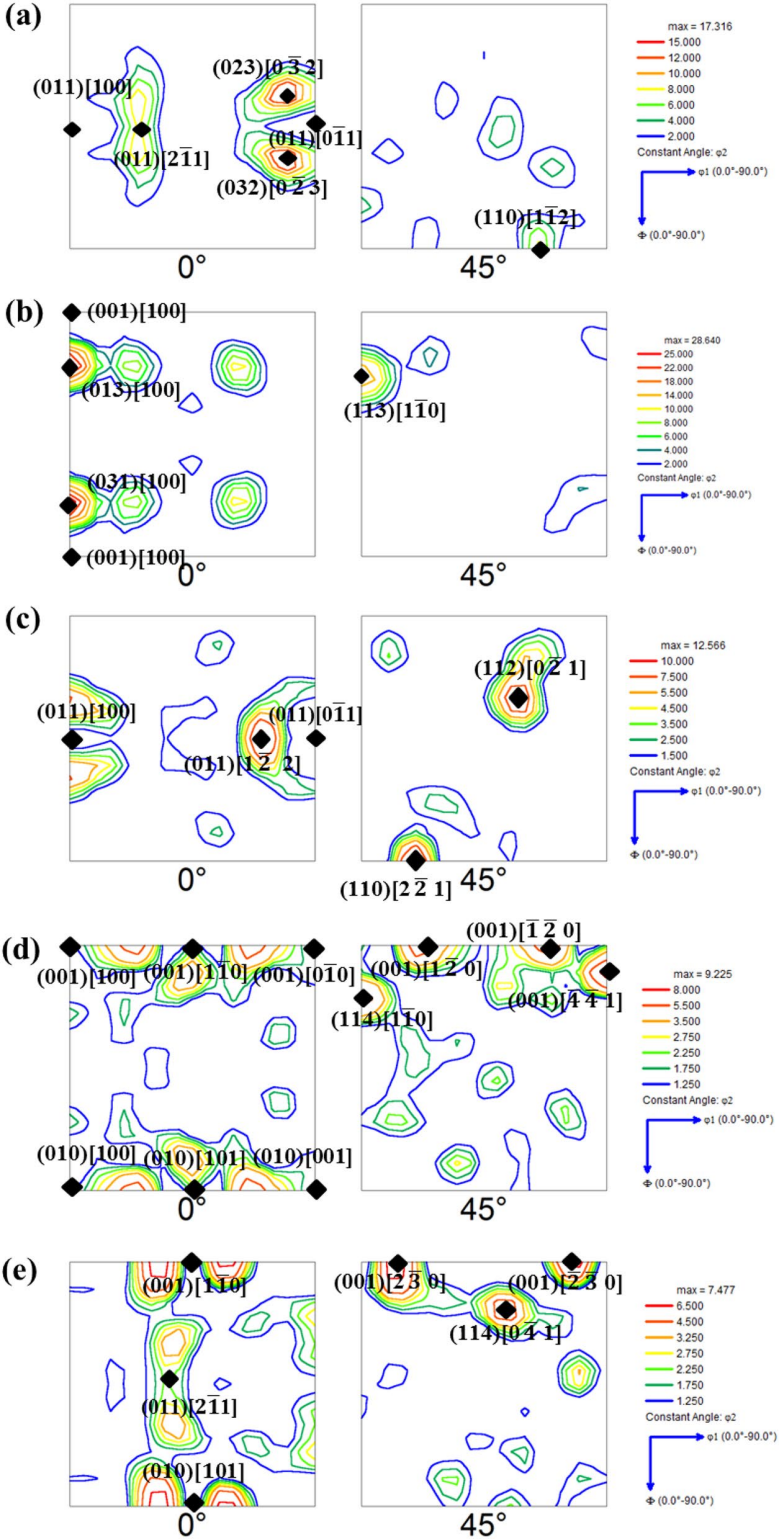
φ_2_ = 0° and 45° sections of the ODF for (**a**) Cast 1; (**b**) Cast 2; (**c**) Cast 3; (**d**) Cast 4 and (**e**) Cast 5.

Figure 5a–e represent the ODF sections of φ_2_ = 0° and 45° for the Cast 1–5 ingots, respectively. The major orientations observed in these ODFs have been marked as well in Fig. 5a–e. As can be seen from Fig. 5a, the as cast structure of Cast 1 consists of columnar grains with crystallographic planes close to {011} parallel to the rolling plane (i.e. perpendicular to ND). This observation is in concurrence with the observation of tilted columnar grains in the IPF given in Fig. 3a. As already discussed, the alignment of the columnar grains is such that they grow opposite to the maximum heat extraction direction. In the process of this growth, the {001} planes being the most loosely packed ones (and with the highest accommodation factor) tend to align perpendicular to the growth direction. Since the maximum heat extraction direction is at an angle to the ND (as shown in Fig. 1c), the {001} planes tend to maintain an angle with the ND and this ultimately leads to development of orientations close to {011} being perpendicular to ND. The major orientations observed are close to Brass component ({011}<112>) and P orientation (Fig. 5a).

In case of Cast 2 ingot, near cube type orientation of the columnar grains is observed. This might have happened due to the effect of magnetic field upon the solidification process. However, it is essential to realize that the solidifying crystals of Fe-Si alloy would start experiencing magnetic field only below the Curie temperature of steel (~ 750 °C) where it is ferromagnetic. Therefore, the effect of magnetic field upon the orientation of the crystals can only be expected when the cast ingot lies in a two phase semi solid zone consisting of solid crystals below Curie temperature (near the mould wall) and surrounding liquid melt (towards center). Under these conditions, the solidified crystals near the mould wall (chilled zone) would tend to have one of the easy magnetization axes ([100]) parallel to the applied field.

Moreover, because of a drastic temperature drop just adjacent to the mould wall, these grains may have one of the loosely packed cube faces, (001) perpendicular to ND.

From these cube on face-oriented crystals, columnar grains opposite to maximum heat extraction directions would grow. This would lead to orientations with [100] || RD, but with crystallographic planes between (001) and (011) perpendicular to ND, as observed in 0° section of Fig. 1b.

In case of Cast 3 ingot, the equiaxed grains (as compared to Cast 1 and Cast 2) with orientations between Goss (i.e. {011}<100>) and Rotated Goss (i.e. {011}<$0\overline{1}1$>) are observed. This indicates that despite of the addition of heterogeneous sites of nucleation to the melt, the as cast structure contains fairly strong texture, which is also supported by the IPF maps (Fig. 3c) and the maximum level of intensity in ODF given in Fig. 5c. The as cast orientations of the ξ fiber (ranging from [100] to [$0\overline{1}1$] parallel to RD) observed in case of Cast 3, indicate that Fe–Co particles by virtue of alignment of the easy magnetization axis along the applied magnetic field have indeed initiated a directional inoculation of the steel melt with cube on face orientations. These nucleated crystals further grew under the effect of fast cooling leading to {011} planes perpendicular to ND. However, these heterogeneously nucleated grains are expected to experience thermo-solutal convection of liquid leading to deviation from ideal <100>|| RD orientations; thus, giving way to near Rotated Goss components. Thus the major observed orientations in Cast 3 ingot are splitted Goss component {011}<100> and P orientation (Fig. 5c).

In case of Cast 4 and 5 ingots, the ODF sections depict randomization of texture due to higher fraction of inoculants (Fig. 5d, e). The grain size in these ingots was also observed to be finer (Fig. 3d, e) as compared to Cast 3, which can again be attributed to addition of higher inoculant fraction. It is worth noting that even though texture randomization has taken place, in case of Cast 4, the orientations are still centred about <001>|| ND fiber (refer Fig. 4a). This indicates the increasing effect of oriented nucleation by directional inoculation over oriented growth by faster cooling rates. The deviation of <100> axes from being parallel to the RD (as would be in case of cube on face orientation) can be attributed to the melt convection either during pouring or due to thermo-solutal gradients. The prominent orientations in both the cases lie within the Cube-Rotated Cube fiber (Fig. 5d, e).

### Microstructure and microtexture of the hot bands

**Figure 6:**
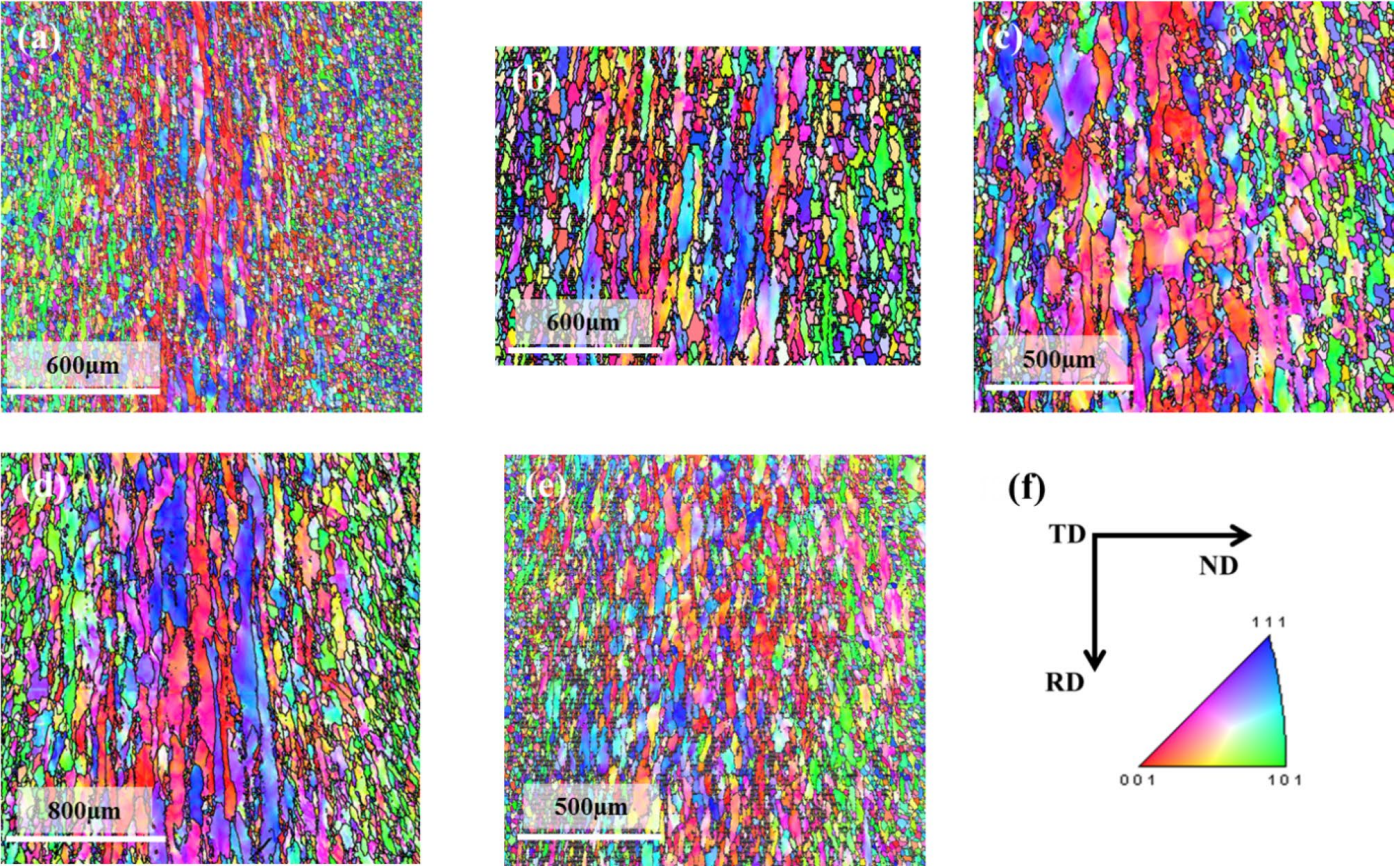
IPF maps of hot bands prepared from (**a**) Cast 1, (**b**) Cast 2, (**c**) Cast 3, (**d**) Cast 4, (**e**) Cast 5 and (**f**) directions of the hot rolled strip with respect to the scanned surface.

The IPF maps acquired on the through thickness ND-RD sections of the hot bands produced from Cast 1–5 ingots are shown in Fig. 6a–e respectively. Figure 6f shows the directions of the hot band with respect to the scanned sections. These IPF maps clearly depict the microstructural and textural gradient which is desirable for the subsequent processing of GO steel^32,34,57,61^. This microstructural gradient consists of recrystallized fine grains at the surface and recovered elongated grains at the center of the strip.

The microstructural gradients although have a similar trend in all the hot rolled strips, yet there were morphological differences between them. Since, neither the alloy composition nor the hot rolling schedule was significantly different in these 5 cases; the differences in the morphology could be attributed to difference in the initial orientations prior to rolling and the subsequent recrystallization kinetics. Therefore, it becomes essential to assess the morphological differences between the different microstructural features of the hot bands in conjunction with the respective microtexture details.

**Figure 7:**
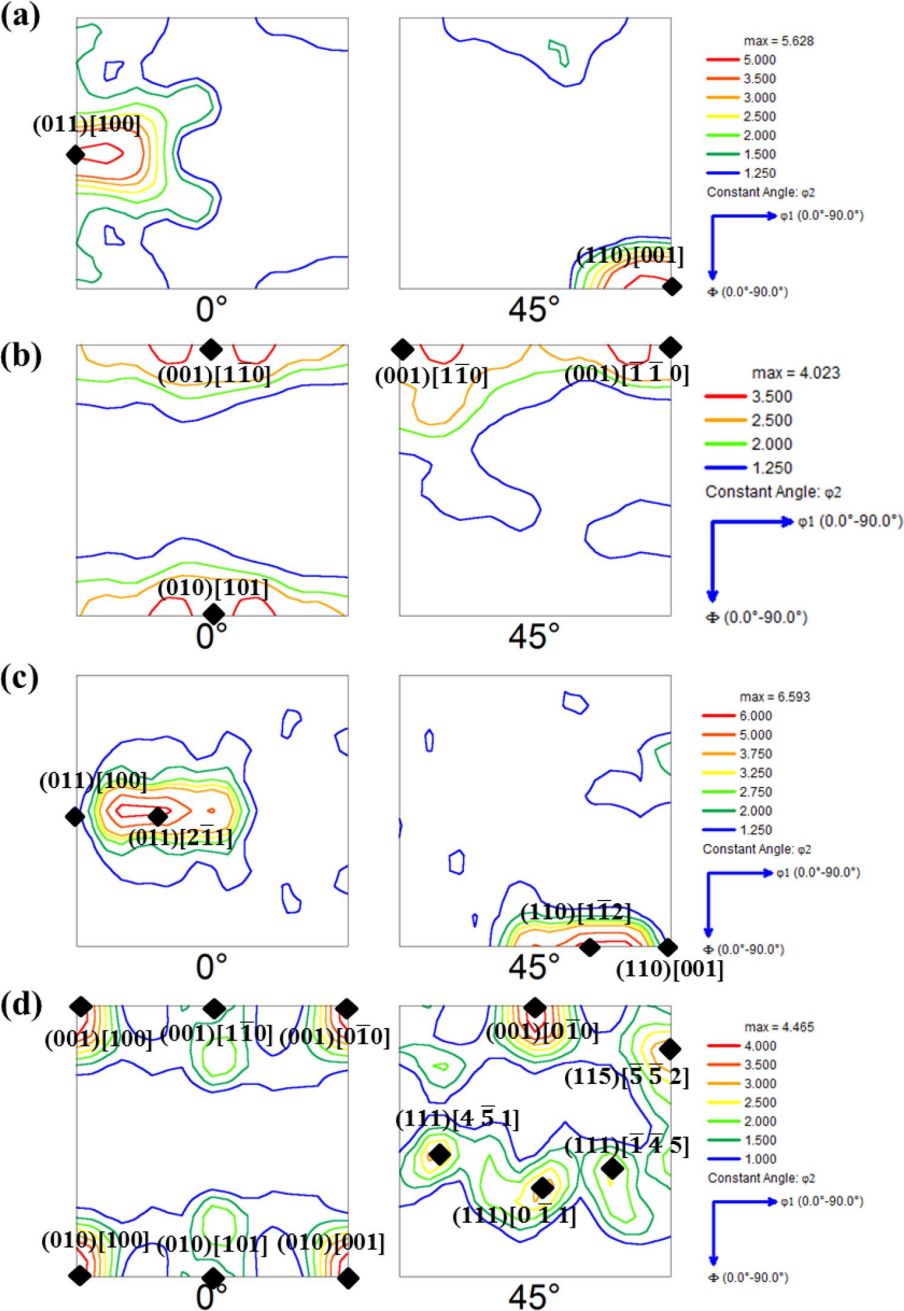
φ_2_ = 0° and 45° sections of hot bands from (**a**) left surface of Cast 1; (**b**) center of Cast 1; (**c**) right surface of Cast 2; (**d**) center of Cast 2.

**Figure 8:**
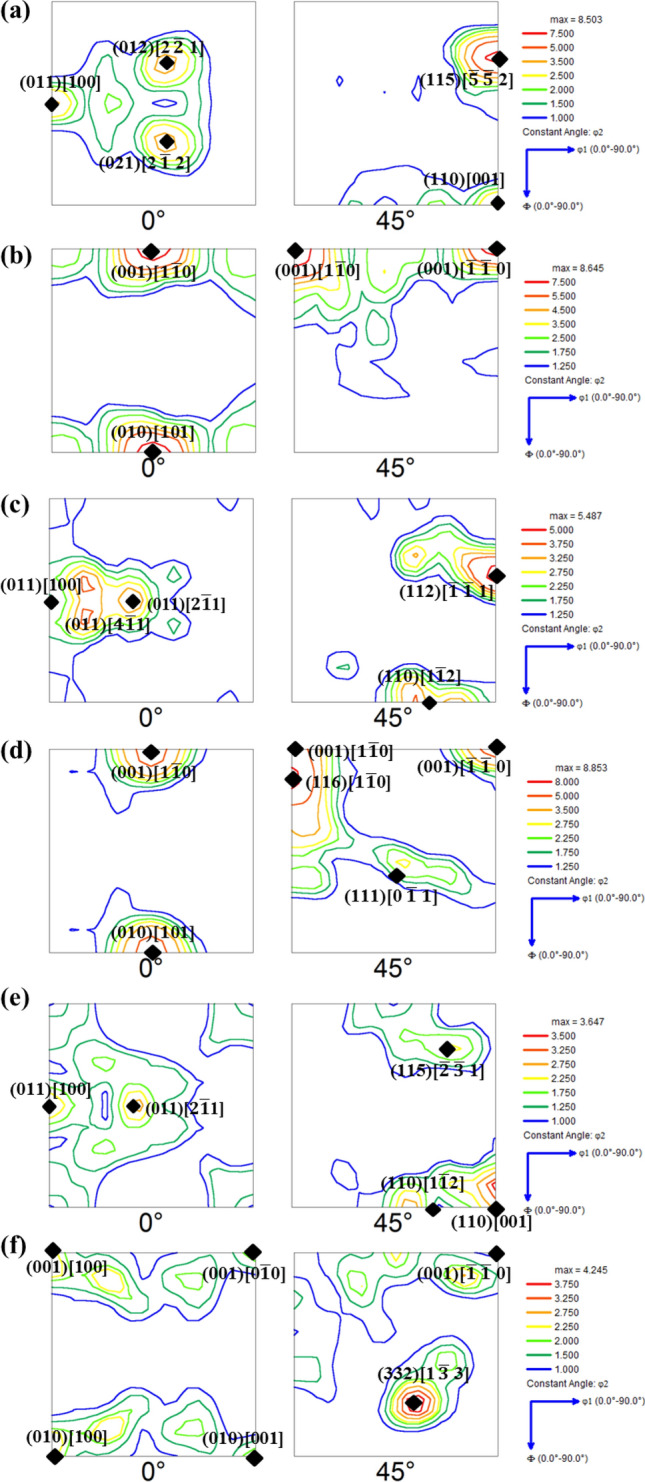
φ_2_ = 0° and 45° ODF sections of hot bands from (**a**) right surface of Cast 3; (**b**) center of Cast 3; (**c**) surface of Cast 4; (**d**) center of Cast 4; (**e**) surface of Cast 5 and (**f**) center of Cast 5.

For this correlation, the ODFs of the surface and center portions of each hot band have been evaluated separately and compared across all the hot bands. The φ_2_ = 0° and 45° sections of ODFs from one of the surfaces and center of the hot bands from Cast 1 and 2 are given in Fig. 7a–d. Similarly, the φ_2_ = 0° and 45° sections of ODFs from one of the surfaces and center of hot bands from Cast 3, 4 and 5 are given in Fig. 8a–f. The major orientations observed in these ODFs of the hot bands (as marked in the respective figures) and their corresponding initial orientations observed in the as cast structure (Fig. 5) are also laid out in Table 3. Before proceeding to the microtexture analysis of the hot rolled strips, it is worth noting that during hot rolling, the surface of the strips experiences extensive shear deformation whereas the center experiences conditions similar to that of plane strain compression^32,33^. Under shear deformation, the grains are expected to rotate about TD and ND or any other axis in between these two, such that the close packed planes (i.e. {011}) become parallel to the rolling plane. In case of PSC, the predominant rotations are about RD and ND. Moreover, deformation has also been shown to be accommodated by rotation taking place within small ‘in band crystal volumes’ about crystallographic axes aligned along TD^31,62^. These rotations are manifested in the microstructure in the form of various fiber texture components; the prominent ones of which are γ fiber (<111>||ND), α fiber (<110>||RD), ξ fiber (<011>||ND) and <001>||ND fiber, etc. The position of these fiber texture components along φ–φ_1_ at constant φ_2_ sections of ODF have been indicated in Fig. 4a, b as well.

**Table 3:** Major texture components observed in the as cast and corresponding hot band microstructures.

Material	Major texture components		
	As cast	Hot band surface	Hot band center
Cast 1	Near Brass ({011}<112>) and near Rotated Goss component ({011}<011>)	Goss orientation ({011}<100>)	Rotated cube orientation ({001}<110>)
Cast 2	Near Cube orientation ({001}<100>)	Near Brass ({011}<112>) orientation	γ fiber components
Cast 3	Near Goss ({011}<100>) and near Rotated Goss component ({011}<011>)	Goss orientation ({011}<100>) and {4 4 11}<11 11 8> orientation	rotated cube orientations ({001}<110>)
Cast 4	Near cube ({001}<100>) and rotated cube orientations ({001}<110>)	Near Goss orientation ({011}<100>) and near Brass ({011}<112>) orientation	γ and α fiber components
Cast 5	Rotated cube orientations ({001}<110>)	Near Goss orientation ({011}<100>) and near Brass ({011}<112>) orientation	γ fiber components and Near cube and rotated cube orientations

It can be seen from Figs. 7a, b and 8a, b, the major texture components in the hot band produced from Cast 1 and 3 are quite similar. The orientations in the surface layers of these hot bands are centred strongly about Goss and other ξ fiber components except for the additional presence of near {4 4 11}<11 11 8> orientations in Cast 3 hot band (Fig. 8a, b). The center of the hot bands from these ingots possess strong <001>||ND fiber texture along with meagre γ fiber components in Cast 1 hot band (Fig. 7a, b).

**Figure 9:**
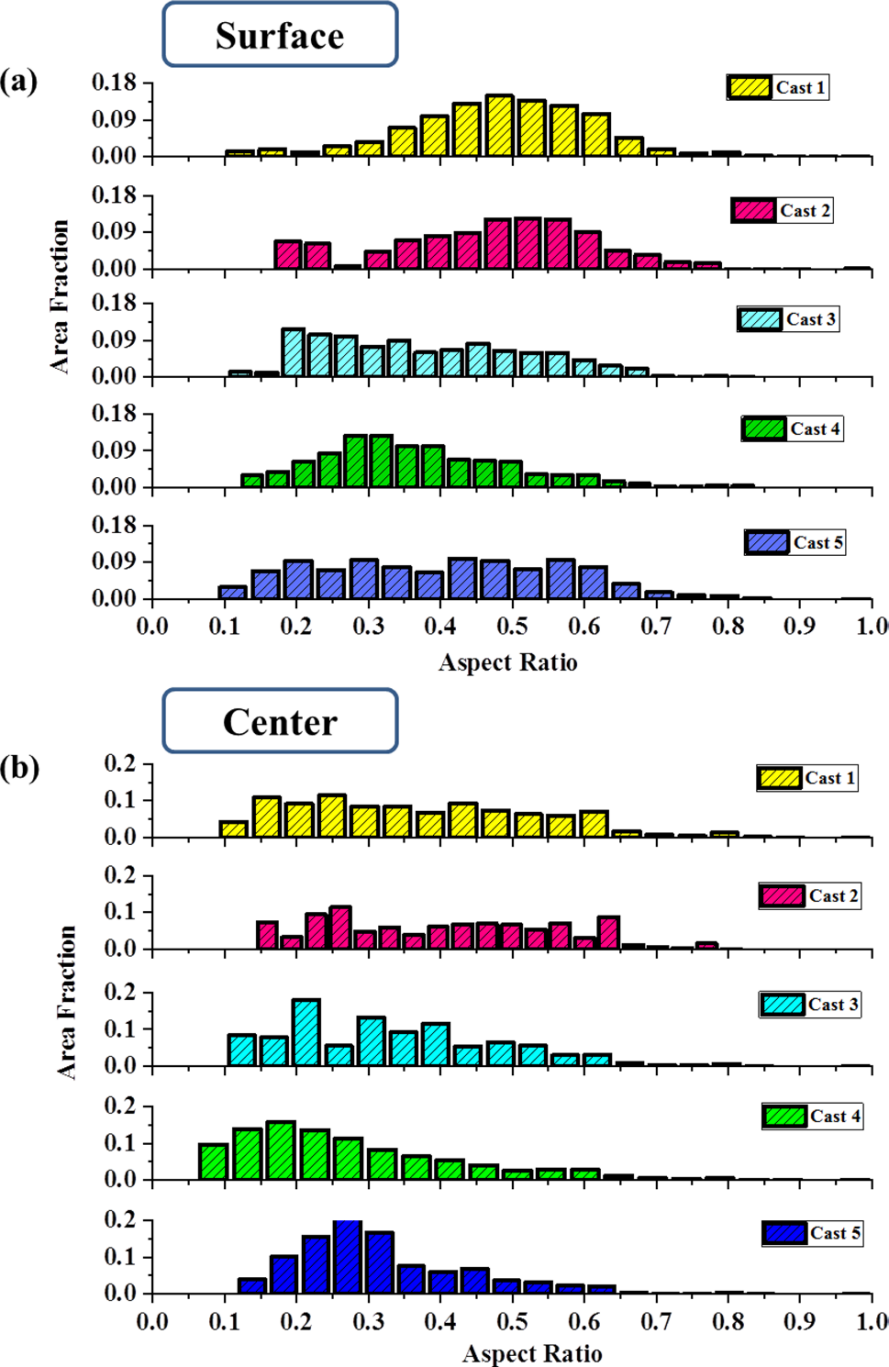
Grain shape aspect ratio distribution in the (**a**) surface layers and (**b**) central layers of all the hot bands produced from different ingots.

In the case of surface of the hot band prepared from Cast 1 and Cast 3 ingots, the initial orientation of {011} planes parallel to the rolling plane, leads to rotation about ND and subsequent Goss texture formation (by repeated recrystallization and deformation)^33^ as observed in Figs. 7a and 8a. These initially {011} parallel to rolling plane oriented grains would recrystallize at relatively later stages as compared to what was expected during the design of the hot rolling schedule (mentioned in “Alloy processing” section). This is due to reduced dislocation activity on these crystallographic orientations^31,62^. Therefore, at the end of the first pass, the surface grains would statically recrystallize during the interpass time, which would further get deformed and partially recrystallized before the last deformation. In the third pass, the surface grains under the effect of previous unrecrystallized state and increased draft undergo dynamic recrystallization leading to formation of very fine recrystallized grains, as observed in Fig. 6a. Despite of the similarity in the initial texture components and hot rolling parameters (and hence, similar aforementioned sequence of deformation and recrystallization), difference in the morphology and grain size of hot bands was observed. The difference in the morphology of grains at hot band surface is more evident from the grain shape aspect ratio charts given in Fig. 9a. The grain shape aspect ratio is measured by fitting an elliptical shape in the grains of the structure and evaluating the ratio of minor to major axis of the ellipse. The maps given in Fig. 9a, b basically depict the grain shape aspect ratio distribution of the surface (the corresponding ODFs of which are given in Figs. 7 and 8) and center of all the hot bands. Figure 9a clearly shows that the aspect ratio of surface grains in Cast 3 hot band is significantly lesser than the Cast 1 hot band, depicting presence of pancaked grains in the former. With similar initial orientations, such contrasting features can be attributed to the difference in the as cast grain size and morphology between the Cast 1 (coarse columnar) and Cast 3 (fine equiaxed) ingots. In case of Cast 1, the first recrystallization would be delayed to later duration of interpass holding (post first pass) due to the coarse microstructure, thereby leading to finer grains before the second pass as compared to Cast 3^48^. These would lead to even finer equiaxed grains in the surface of hot band produced from Cast 1 as compared to that of Cast 3 (refer Fig. 6a, c). The aspect ratio distribution of Cast 1 and 3 hot bands given in Fig. 9a confirms the hypothesized sequence of deformation and recrystallization as well.

In the central layers of the hot band prepared from Cast 1 ingot, the near Goss oriented grains present in the as cast structure rotate to γ fiber components which are the stable end orientations under PSC condition^29,31,33^ as observed in the φ_2_ = 45° sections of Fig. 7b. Moreover, the initial near rotated Goss orientations present in Cast 1 and Cast 3 ingots, under PSC would develop near cube orientations in localized crystal volumes which evolve as recrystallized cube oriented grains as well^62^. These can further lead to both γ fiber components and rotated cube orientations as prominently observed in Figs. 7b and 8b respectively. As already discussed, the difference in the initial grain size of Cast 1 and Cast 3 leads to the delayed recrystallization of the central layers in case of hot band of Cast1 as compared to that of Cast 3. This, further under deformation in subsequent pass leads to grains with lower aspect ratio (in the range of 0–0.2) to be more prominently present in the central layers of Cast 1 (Figs. 6a and 9b) as compared to that of Cast 3 (Figs. 6c and 9b).

Similarity in the hot band texture can also be observed amongst Cast 2, 4 and 5. The surface layers of the hot bands from these ingots exhibit strong Goss and ξ fiber components, as can be seen from Figs. 7c and 8c, e. In contrast to the surface, the center portions of the hot bands from these ingots possess strong γ and α fiber texture. In case of Cast 2, 4 and 5, the initially near cube oriented grains would rotate about TD and ND (or any other direction in between) leading to strong near Goss texture or other ξ fiber components in the surface layers as observed in φ_2_ = 0° sections of Figs. 7c, and 8c, e, respectively. The presence of these ξ fiber components confirm that the initial rotation takes place about TD or other directions close to TD so that the cube orientations attain {011} plane parallel to RD. The subsequent rotations take place about ND as these ξ fiber components consist of stable end orientations under shear deformation. The rotation about TD is also confirmed from the presence of orientations near another shear induced component, {4 4 11}<11 11 8> , as observed in the φ_2_ = 45° section of Figs. 7c, and 8c, e.

The strong γ and α fiber components in the central layers of the hot bands from Cast 2, 4 and 5 owe their origin to the initial near cube orientations, which under PSC would easily rotate about ND to yield stable rotated cube components and about RD to γ fiber components^42^. The γ fiber texture, although is observed to be stronger in case of Cast 2 hot band (Fig. 7d) as compared to Cast 4 or 5 (Fig. 8d, f), yet the morphological gradient for further processing seems to be more suitable in case of Cast 4 and 5 hot bands (Fig. 6c, d). This is also clearly evident from the grain shape aspect ratio distribution given in Fig. 9b where the central layers of Cast 2 hot band are shown to possess high aspect ratio grains in contrary to Cast 4 and 5 hot bands. The underlying reason for this difference in morphological gradient is the combination of starting texture component and grain size, which in case of Cast 2 are near cube and coarse columnar respectively. These coarse columnar near cube oriented grains can easily rotate about ND to stable end rotated cube components which have highly sluggish recrystallization kinetics^63^. This leads to delayed recrystallization in the later stages of interpass holding post first pass of hot rolling, static recovery post the second pass followed by partial dynamic recrystallization in the final pass (refer Fig. 6b). On the contrary, due to the fine equiaxed grains present in the as cast structure of Cast 4 and 5, the recrystallization takes place at an earlier stage followed by growth, post the first pass of hot rolling. These would undergo dynamic and static recovery post the second and third pass, ultimately leading to recovered grain in the center as observed in Fig. 6d, e.

Thus, the hot bands from Cast 4 and 5 consist of fine Goss to Brass ({011}<211>) oriented grains in the surface and elongated recovered γ and α fiber components, which is most suitable for further processing of GO steels.

**Figure 10:**
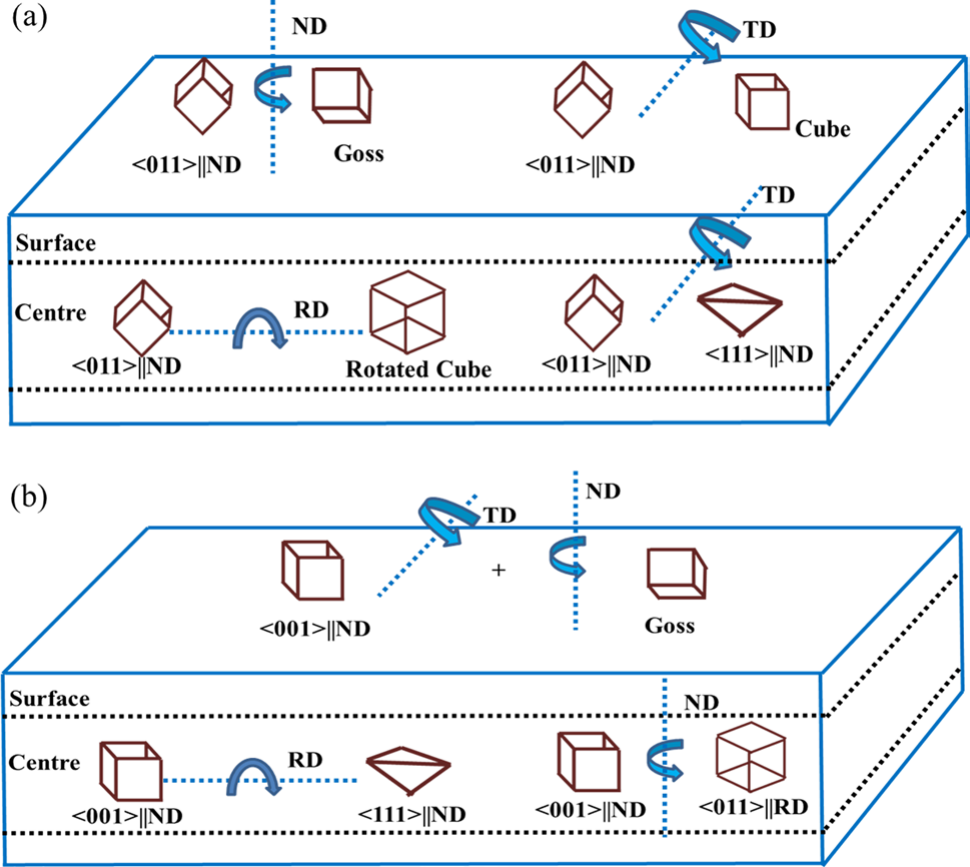
Schematic representation of the major texture components of the hot band and the corresponding plausible evolution from the initial orientations for (**a**) Cast 1 and 3; (**b**) Cast 2, 4 and 5.

The major texture components of the hot band and the corresponding plausible evolution from the initial orientations of each ingot are shown schematically in Fig. 10a for Cast 1, Cast 3 and Fig. 10b for Cast 2, 4 and 5.

## Conclusion

In the present work, a novel technique of directional inoculation has been devised and applied for production of hot bands with microstructural and textural gradient suitable for GO steel production. The target was to cast ingots under the effect of directional inoculation so as to begin with an as cast thin slab containing Goss oriented components. The target was set so, because the Goss components are expected to provide potential nuclei (Goss grains) as well as suitable surrounding texture (γ and α fiber components) for abnormal grain growth. The design of the directional inoculation technique relied upon the use of soft magnetic inoculants for providing oriented nucleation under the effect of external magnetic field, as well as upon the fast cooling rates for providing oriented growth. Ingots were cast under different conditions and the solidification texture was analysed in light of the effect of various involved parameters. It was observed that the directional inoculation technique was indeed capable of inducing near Goss oriented components in the as cast structure when the inoculants were added at an optimal amount. Higher addition of inoculants led to finer equiaxed grains in the as cast structure with cubic orientations. The hot rolling of these ingots was carried out and it was observed that the ingots with cube texture components and fine grains evolved into the most suitable hot band microstructure with the requisite microstructural and microtextural gradient. The developed hot band textures in case of each ingot was analysed in terms of different plausible rotations of crystal during deformation. It was observed that during hot rolling, the most predominant rotations for the surface of hot band take place about the ND and TD (or any other direction in between). On the contrary, for the central layers, the most prominent rotations are about all the three ND, RD and TD, depending upon the initial orientation.
